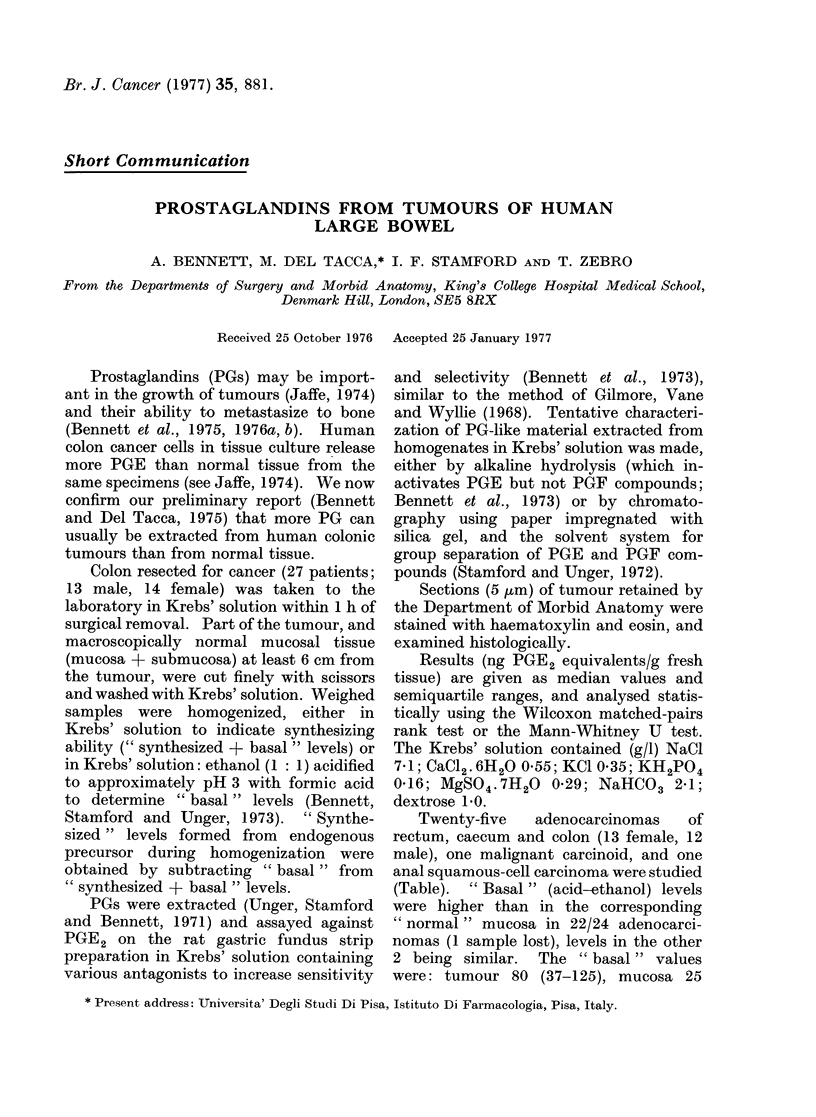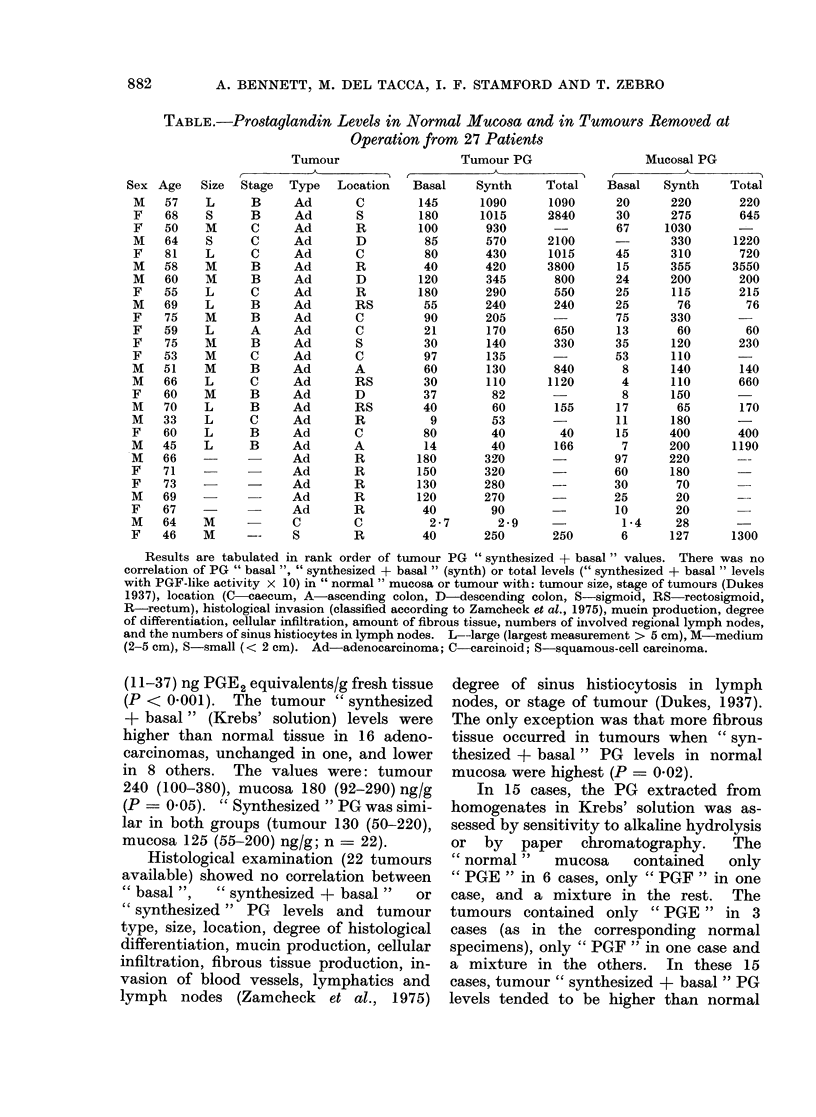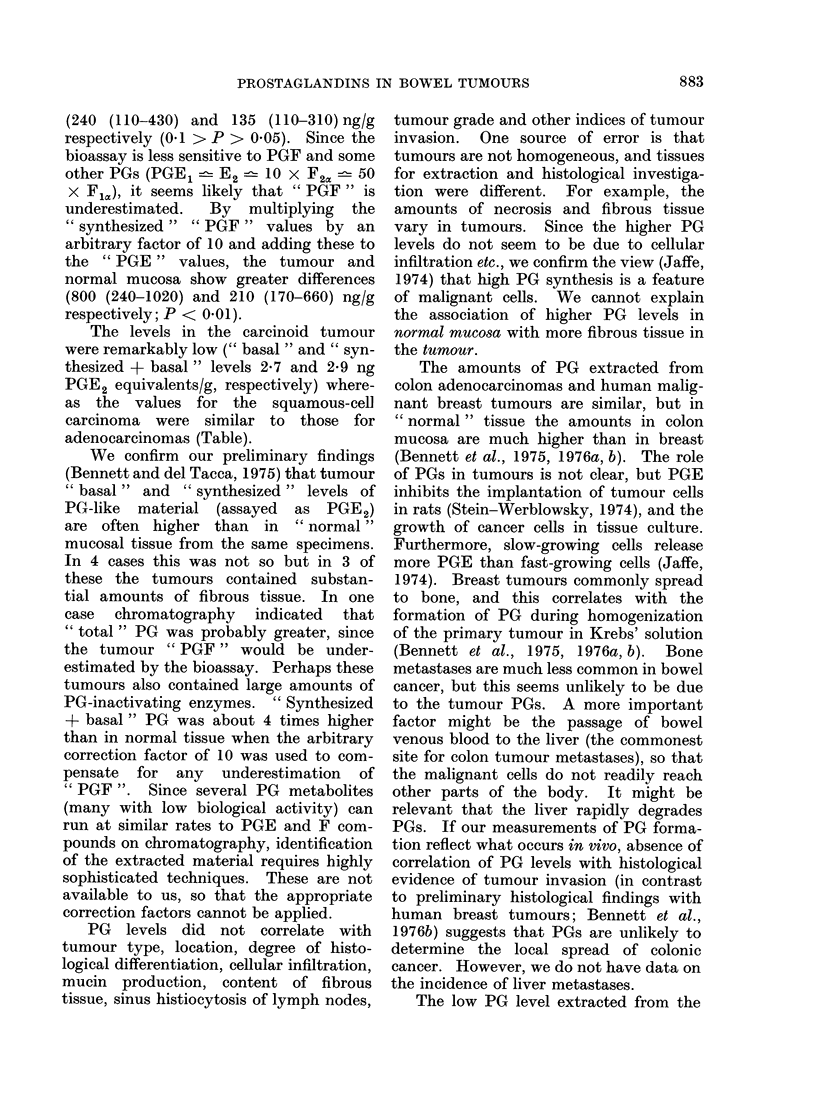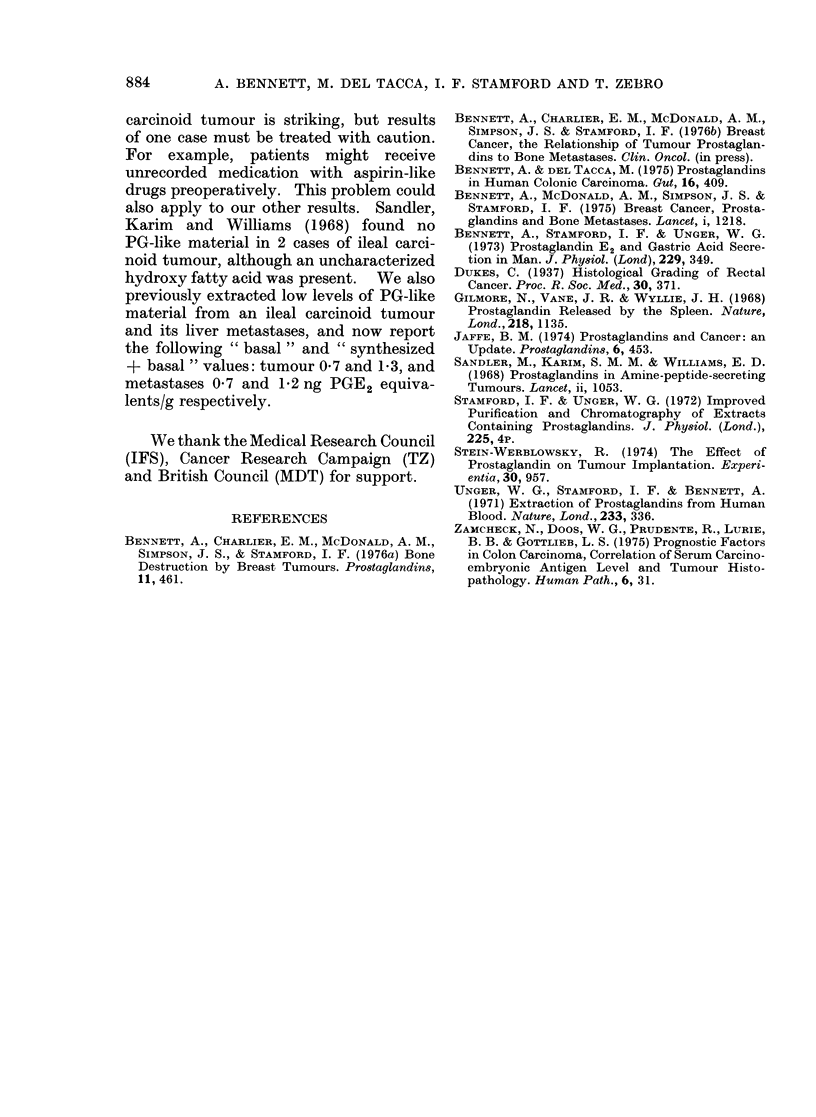# Prostaglandins from tumours of human large bowel.

**DOI:** 10.1038/bjc.1977.132

**Published:** 1977-06

**Authors:** A. Bennett, M. D. Tacca, I. F. Stamford, T. Zebro


*Br. J. Cancer* (1977) **35**, 881.

***Short Communication***

### PROSTAGLANDINS FROM TUMOURS OF HUMAN LARGE BOWEL

A. BENNETT, M. DEL TACCA,* I. F. STAMFORD AND T. ZEBRO

*From the Departments of Surgery and Morbid Anatomy, King's College Hospital Medical School, Denmark Hill, London, SE5 8RX*

Received 25 October 1976 Accepted 25 January 1977

Prostaglandins (PGs) may be important in the growth of tumours (Jaffe, 1974) and their ability to metastasize to bone (Bennett *et al.*, 1975, 1976*a*, *b*). Human colon cancer cells in tissue culture release more PGE than normal tissue from the same specimens (see Jaffe, 1974). We now confirm our preliminary report (Bennett and Del Tacca, 1975) that more PG can usually be extracted from human colonic tumours than from normal tissue.

Colon resected for cancer (27 patients; 13 male, 14 female) was taken to the laboratory in Krebs' solution within 1 h of surgical removal. Part of the tumour, and macroscopically normal mucosal tissue (mucosa + submucosa) at least 6 cm from the tumour, were cut finely with scissors and washed with Krebs' solution. Weighed samples were homogenized, either in Krebs' solution to indicate synthesizing ability (" synthesized + basal " levels) or in Krebs' solution: ethanol (1 : 1) acidified to approximately pH 3 with formic acid to determine " basal " levels (Bennett, Stamford and Unger, 1973). " Synthesized " levels formed from endogenous precursor during homogenization were obtained by subtracting " basal " from " synthesized + basal " levels.

PGs were extracted (Unger, Stamford and Bennett, 1971) and assayed against $PGE_2$ on the rat gastric fundus strip preparation in Krebs' solution containing various antagonists to increase sensitivity and selectivity (Bennett *et al.*, 1973), similar to the method of Gilmore, Vane and Wyllie (1968). Tentative characterization of PG-like material extracted from homogenates in Krebs' solution was made, either by alkaline hydrolysis (which inactivates PGE but not PGF compounds; Bennett *et al.*, 1973) or by chromatography using paper impregnated with silica gel, and the solvent system for group separation of PGE and PGF compounds (Stamford and Unger, 1972).

Sections (5 $\mu$m) of tumour retained by the Department of Morbid Anatomy were stained with haematoxylin and eosin, and examined histologically.

Results (ng $PGE_2$ equivalents/g fresh tissue) are given as median values and semiquartile ranges, and analysed statistically using the Wilcoxon matched-pairs rank test or the Mann-Whitney U test. The Krebs' solution contained (g/l) NaCl 7·1; $CaCl_2.6H_2O$ 0·55; KCl 0·35; $KH_2PO_4$ 0·16; $MgSO_4.7H_2O$ 0·29; $NaHCO_3$ 2·1; dextrose 1·0.

Twenty-five adenocarcinomas of rectum, caecum and colon (13 female, 12 male), one malignant carcinoid, and one anal squamous-cell carcinoma were studied (Table). " Basal " (acid–ethanol) levels were higher than in the corresponding " normal " mucosa in 22/24 adenocarcinomas (1 sample lost), levels in the other 2 being similar. The " basal " values were: tumour 80 (37–125), mucosa 25

* Present address: Universita' Degli Studi Di Pisa, Istituto Di Farmacologia, Pisa, Italy.

TABLE.—*Prostaglandin Levels in Normal Mucosa and in Tumours Removed at Operation from 27 Patients*

| | | | Tumour | | | Tumour PG | | | Mucosal PG | | |
|---|---|---|---|---|---|---|---|---|---|---|---|
| Sex | Age | Size | Stage | Type | Location | Basal | Synth | Total | Basal | Synth | Total |
| M | 57 | L | B | Ad | C | 145 | 1090 | 1090 | 20 | 220 | 220 |
| F | 68 | S | B | Ad | S | 180 | 1015 | 2840 | 30 | 275 | 645 |
| F | 50 | M | C | Ad | R | 100 | 930 | — | 67 | 1030 | — |
| M | 64 | S | C | Ad | D | 85 | 570 | 2100 | — | 330 | 1220 |
| F | 81 | L | C | Ad | C | 80 | 430 | 1015 | 45 | 310 | 720 |
| M | 58 | M | B | Ad | R | 40 | 420 | 3800 | 15 | 355 | 3550 |
| M | 60 | M | B | Ad | D | 120 | 345 | 800 | 24 | 200 | 200 |
| F | 55 | L | C | Ad | R | 180 | 290 | 550 | 25 | 115 | 215 |
| M | 69 | L | B | Ad | RS | 55 | 240 | 240 | 25 | 76 | 76 |
| F | 75 | M | B | Ad | C | 90 | 205 | — | 75 | 330 | — |
| F | 59 | L | A | Ad | C | 21 | 170 | 650 | 13 | 60 | 60 |
| F | 75 | M | B | Ad | S | 30 | 140 | 330 | 35 | 120 | 230 |
| F | 53 | M | C | Ad | C | 97 | 135 | — | 53 | 110 | — |
| M | 51 | M | B | Ad | A | 60 | 130 | 840 | 8 | 140 | 140 |
| M | 66 | L | C | Ad | RS | 30 | 110 | 1120 | 4 | 110 | 660 |
| F | 60 | M | B | Ad | D | 37 | 82 | — | 8 | 150 | — |
| M | 70 | L | B | Ad | RS | 40 | 60 | 155 | 17 | 65 | 170 |
| M | 33 | L | C | Ad | R | 9 | 53 | — | 11 | 180 | — |
| F | 60 | L | B | Ad | C | 80 | 40 | 40 | 15 | 400 | 400 |
| M | 45 | L | B | Ad | A | 14 | 40 | 166 | 7 | 200 | 1190 |
| M | 66 | — | — | Ad | R | 180 | 320 | — | 97 | 220 | — |
| F | 71 | — | — | Ad | R | 150 | 320 | — | 60 | 180 | — |
| F | 73 | — | — | Ad | R | 130 | 280 | — | 30 | 70 | — |
| M | 69 | — | — | Ad | R | 120 | 270 | — | 25 | 20 | — |
| F | 67 | — | — | Ad | R | 40 | 90 | — | 10 | 20 | — |
| M | 64 | M | — | C | C | 2·7 | 2·9 | — | 1·4 | 28 | — |
| F | 46 | M | — | S | R | 40 | 250 | 250 | 6 | 127 | 1300 |

Results are tabulated in rank order of tumour PG " synthesized + basal " values. There was no correlation of PG " basal ", " synthesized + basal " (synth) or total levels (" synthesized + basal " levels with PGF-like activity × 10) in " normal " mucosa or tumour with: tumour size, stage of tumours (Dukes 1937), location (C—caecum, A—ascending colon, D—descending colon, S—sigmoid, RS—rectosigmoid, R—rectum), histological invasion (classified according to Zamcheck *et al.*, 1975), mucin production, degree of differentiation, cellular infiltration, amount of fibrous tissue, numbers of involved regional lymph nodes, and the numbers of sinus histiocytes in lymph nodes. L—large (largest measurement > 5 cm), M—medium (2–5 cm), S—small (< 2 cm). Ad—adenocarcinoma; C—carcinoid; S—squamous-cell carcinoma.

(11–37) ng $PGE_2$ equivalents/g fresh tissue ($P < 0{\cdot}001$). The tumour " synthesized + basal " (Krebs' solution) levels were higher than normal tissue in 16 adenocarcinomas, unchanged in one, and lower in 8 others. The values were: tumour 240 (100–380), mucosa 180 (92–290) ng/g ($P = 0{\cdot}05$). " Synthesized " PG was similar in both groups (tumour 130 (50–220), mucosa 125 (55–200) ng/g; n = 22).

Histological examination (22 tumours available) showed no correlation between " basal ", " synthesized + basal " or " synthesized " PG levels and tumour type, size, location, degree of histological differentiation, mucin production, cellular infiltration, fibrous tissue production, invasion of blood vessels, lymphatics and lymph nodes (Zamcheck *et al.*, 1975) degree of sinus histiocytosis in lymph nodes, or stage of tumour (Dukes, 1937). The only exception was that more fibrous tissue occurred in tumours when " synthesized + basal " PG levels in normal mucosa were highest ($P = 0{\cdot}02$).

In 15 cases, the PG extracted from homogenates in Krebs' solution was assessed by sensitivity to alkaline hydrolysis or by paper chromatography. The " normal " mucosa contained only " PGE " in 6 cases, only " PGF " in one case, and a mixture in the rest. The tumours contained only " PGE " in 3 cases (as in the corresponding normal specimens), only " PGF " in one case and a mixture in the others. In these 15 cases, tumour " synthesized + basal " PG levels tended to be higher than normal

(240 (110–430) and 135 (110–310) ng/g respectively ($0{\cdot}1 > P > 0{\cdot}05$). Since the bioassay is less sensitive to PGF and some other PGs ($PGE_1 \doteqdot E_2 \doteqdot 10 \times F_{2\alpha} \doteqdot 50 \times F_{1\alpha}$), it seems likely that " PGF " is underestimated. By multiplying the " synthesized " " PGF " values by an arbitrary factor of 10 and adding these to the " PGE " values, the tumour and normal mucosa show greater differences (800 (240–1020) and 210 (170–660) ng/g respectively; $P < 0{\cdot}01$).

The levels in the carcinoid tumour were remarkably low (" basal " and " synthesized + basal " levels 2·7 and 2·9 ng $PGE_2$ equivalents/g, respectively) whereas the values for the squamous-cell carcinoma were similar to those for adenocarcinomas (Table).

We confirm our preliminary findings (Bennett and del Tacca, 1975) that tumour " basal " and " synthesized " levels of PG-like material (assayed as $PGE_2$) are often higher than in " normal " mucosal tissue from the same specimens. In 4 cases this was not so but in 3 of these the tumours contained substantial amounts of fibrous tissue. In one case chromatography indicated that " total " PG was probably greater, since the tumour " PGF " would be underestimated by the bioassay. Perhaps these tumours also contained large amounts of PG-inactivating enzymes. " Synthesized + basal " PG was about 4 times higher than in normal tissue when the arbitrary correction factor of 10 was used to compensate for any underestimation of " PGF ". Since several PG metabolites (many with low biological activity) can run at similar rates to PGE and F compounds on chromatography, identification of the extracted material requires highly sophisticated techniques. These are not available to us, so that the appropriate correction factors cannot be applied.

PG levels did not correlate with tumour type, location, degree of histological differentiation, cellular infiltration, mucin production, content of fibrous tissue, sinus histiocytosis of lymph nodes, tumour grade and other indices of tumour invasion. One source of error is that tumours are not homogeneous, and tissues for extraction and histological investigation were different. For example, the amounts of necrosis and fibrous tissue vary in tumours. Since the higher PG levels do not seem to be due to cellular infiltration *etc.*, we confirm the view (Jaffe, 1974) that high PG synthesis is a feature of malignant cells. We cannot explain the association of higher PG levels in *normal mucosa* with more fibrous tissue in the *tumour*.

The amounts of PG extracted from colon adenocarcinomas and human malignant breast tumours are similar, but in " normal " tissue the amounts in colon mucosa are much higher than in breast (Bennett *et al.*, 1975, 1976*a*, *b*). The role of PGs in tumours is not clear, but PGE inhibits the implantation of tumour cells in rats (Stein–Werblowsky, 1974), and the growth of cancer cells in tissue culture. Furthermore, slow-growing cells release more PGE than fast-growing cells (Jaffe, 1974). Breast tumours commonly spread to bone, and this correlates with the formation of PG during homogenization of the primary tumour in Krebs' solution (Bennett *et al.*, 1975, 1976*a*, *b*). Bone metastases are much less common in bowel cancer, but this seems unlikely to be due to the tumour PGs. A more important factor might be the passage of bowel venous blood to the liver (the commonest site for colon tumour metastases), so that the malignant cells do not readily reach other parts of the body. It might be relevant that the liver rapidly degrades PGs. If our measurements of PG formation reflect what occurs *in vivo*, absence of correlation of PG levels with histological evidence of tumour invasion (in contrast to preliminary histological findings with human breast tumours; Bennett *et al.*, 1976*b*) suggests that PGs are unlikely to determine the local spread of colonic cancer. However, we do not have data on the incidence of liver metastases.

The low PG level extracted from the carcinoid tumour is striking, but results of one case must be treated with caution. For example, patients might receive unrecorded medication with aspirin-like drugs preoperatively. This problem could also apply to our other results. Sandler, Karim and Williams (1968) found no PG-like material in 2 cases of ileal carcinoid tumour, although an uncharacterized hydroxy fatty acid was present. We also previously extracted low levels of PG-like material from an ileal carcinoid tumour and its liver metastases, and now report the following " basal " and " synthesized + basal " values: tumour 0·7 and 1·3, and metastases 0·7 and 1·2 ng $PGE_2$ equivalents/g respectively.

We thank the Medical Research Council (IFS), Cancer Research Campaign (TZ) and British Council (MDT) for support.

### REFERENCES

Bennett, A., Charlier, E. M., McDonald, A. M., Simpson, J. S., & Stamford, I. F. (1976*a*) Bone Destruction by Breast Tumours. *Prostaglandins*, **11**, 461.

Bennett, A., Charlier, E. M., McDonald, A. M., Simpson, J. S. & Stamford, I. F. (1976*b*) Breast Cancer, the Relationship of Tumour Prostaglandins to Bone Metastases. *Clin. Oncol.* (in press).

Bennett, A. & del Tacca, M. (1975) Prostaglandins in Human Colonic Carcinoma. *Gut*, **16**, 409.

Bennett, A., McDonald, A. M., Simpson, J. S. & Stamford, I. F. (1975) Breast Cancer, Prostaglandins and Bone Metastases. *Lancet*, i, 1218.

Bennett, A., Stamford, I. F. & Unger, W. G. (1973) Prostaglandin $E_2$ and Gastric Acid Secretion in Man. *J. Physiol.* (*Lond*), **229**, 349.

Dukes, C. (1937) Histological Grading of Rectal Cancer. *Proc. R. Soc. Med.*, **30**, 371.

Gilmore, N., Vane, J. R. & Wyllie, J. H. (1968) Prostaglandin Released by the Spleen. *Nature, Lond.*, **218**, 1135.

Jaffe, B. M. (1974) Prostaglandins and Cancer: an Update. *Prostaglandins*, **6**, 453.

Sandler, M., Karim, S. M. M. & Williams, E. D. (1968) Prostaglandins in Amine-peptide-secreting Tumours. *Lancet*, ii, 1053.

Stamford, I. F. & Unger, W. G. (1972) Improved Purification and Chromatography of Extracts Containing Prostaglandins. *J. Physiol.* (*Lond.*), **225**, 4P.

Stein-Werblowsky, R. (1974) The Effect of Prostaglandin on Tumour Implantation. *Experientia*, **30**, 957.

Unger, W. G., Stamford, I. F. & Bennett, A. (1971) Extraction of Prostaglandins from Human Blood. *Nature, Lond.*, **233**, 336.

Zamcheck, N., Doos, W. G., Prudente, R., Lurie, B. B. & Gottlieb, L. S. (1975) Prognostic Factors in Colon Carcinoma, Correlation of Serum Carcinoembryonic Antigen Level and Tumour Histopathology. *Human Path.*, **6**, 31.